# Supplementary material for: Association of CYP2C19, CYP2D6 and CYP3A4 Genetic Variants on Primaquine Hemolysis in G6PD-Deficient Patients
Source: Pathogens. 2023 Jun 30;12(7):895. doi: 10.3390/pathogens12070895 (PMC10384057; doi:10.3390/pathogens12070895)
Supplement: Supplementary file 1 [file pathogens-12-00895-s001.zip › pathogens-2410502-supplementary.pdf]

Table S1. Hemolytic aspect in G6PDd individuals with CYP phenotypes and genotypes.

Characteristics	*CYP2C19* (N= 13)				*CYP2D6* (N= 13)				*CYP3A4* (N= 13)		
	gNM	gIM	gRM	p value	gNM	gIM	gUM	p value	*1/*1	*1/*1B	p value
A376G, n/N (%)	1 (16.7)	0 (0.0)	1 (25.0)		2 (18.2)	0 (0.0)	0 (0.0		0 (0.0)	2 (100.0)	
G202A/A376G, n/N (%)	4 (66.7)	4 (100.0)	2 (50.0)	0.860	8 (72.7)	1 (100.0)	1 (100.0)	>0.999	10 (90.9)	0 (0.0)	**0.013**
C563T, n/N (%)	1 (16.7)	0 (0.0)	0 (0.0)		1 (9.1)	0 (0.0)	0 (0.0)		1 (9.1)	0 (0.0)	
Mild anemia (Hb), n/N (%)	0 (0.0)	0 (0.0)	0 (0.0)		0 (0.0)	0 (0.0)	0 (0.0)		0 (0.0)	0 (0.0)	
Moderate Anemia (Hb), n/N (%)	4 (66.7)	1 (25.0)	1 (33.3)	0.493	5 (45.4)	1 (100.0)	0 (0.0)	>0.999	5 (45.4)	1 (50.0)	0.731
Severe anemia (Hb), n/N (%)	2 (33.3)	3 (75.0)	2 (66.7)		6 (54.5)	-	1 (100.0)		6 (54.5)	1 (50.0)	
TB (mg/dL), median (IQR)	2.6 (1.5-4.2)	1.1 (0.8-1.7)	4.4 (2.4-8.8)	0.0614	2.3 (1.2-3.8)	0.8 (-)	8.8 (-)	**0.0048**	2.1 (1.0-3.6)	3.5 (2.6-4.4)	0.2827
DB (mg/dL), median (IQR)	0.5 (0.3-0.8)	0.3 (0.3-0.8)	1.3 (1.0-5.3)	**0.0494**	0.5 (0.4-1.0)	0.3 (-)	5.3 (-)	**<0.0001**	0.4 (0.3-1.0)	0.9 (0.5-1.3)	0.3277
IB (mg/dL), median (IQR)	2.1 (1.1-3.5)	0.7 (0.4-1.0)	3.1 (1.4-3.5)	0.0615	1.6 (0.8-3.1)	0.5 (-)	5.3 (-)	**0.0392**	1.2 (0.6-3.2)	2.6 (2.1-3.1)	0.3902
SGOT (U/L), median (IQR)	53 (35.7-83.2)	76.5 (34.7-121.3)	99 (62- 120)	0.4295	62 (36-114)	45 (-)	99 (-)	0.5901	61 (36-101)	88 (62-114)	0.4298
SGPT (U/L), median (IQR)	57.5 (44.7-60.2)	39 (15.7-61.5)	154 (69-204)	**0.0052**	59 (41-66)	56 (-)	154 (-)	0.5513	56 (41-66)	64.5 (60-96)	0.3237
LDH (U/L), median (IQR)	2,119 (878.5-4,555)	1,251 (574-2,422)	1,424 (772-2,044)	0.5023	1424 (835-2786)	859 (-)	2044 (-)	0.7818	1,251 (859-2,119)	3692 (1,424-5,960)	0.2386
Glucose (mg/dL), mean (SD)	106.4 (26.8)	128.5 (53.0)	126.7 (16.6)	0.5647	117.6 (28.1)	84 (-)	144 (-)	0.3689	117.5 (30.2)	111 (-)	0.8614

Creatinine (mg/dL), median (IQR)	1.0 (0.7-1.3)	2.3 (0.3-5.9)	0.8 (0.7-9.7)	0.9864	1.0 (0.7-1.6)	0.5 (-)	9.7 (-)	0.0769	1.0 (0.7-4.0)	1.0 (0.7-1.3)	0.7669
Urea (mg/dL), median (IQR)	43.5 (33.2-66)	92 (18-212.5)	41 (28-277)	0.4355	43 (28-75)	10 (-)	277 (-)	**0.0256**	43 (18-166)	58 (41-75)	0.7665
Hemoglobinuria, n/N (%)	5 (83.3)	2 (100.0)	1 (50.0)	0.667	7 (77.8)	1 (100.0)	-	0.800	6 (75.0)	2 (100.0)	0.622
Proteinuria, n/N (%)	4 (66.7)	1 (50.0)	1 (50.0)	>0.999	6 (66.7)	-	-	0.400	4 (50.0)	2 (100.0)	0.333
Bilirubinuria, n/N (%)	3 (50.0)	-	-	0.300	3 (33.3)	-	-	0.700	2 (25.0)	1 (50.0)	0.533

Abbreviations: Hb= hemoglobin, TB= total bilirubin, DB= direct bilirubin, IB= indirect bilirubin, LDH= lactic dehydrogenase, G202A/A376G= African A-; A376G= African A+; C563T= Mediterranean, gNM= normal metabolizer, gIM= intermediate metabolizer, gRM= rapid metabolizer, gUM= ultra-rapid metabolizer, SD= standard deviation, IQR= Interquartile range. Symbol * refers to the star allele.

Table S2. Hemolytic aspect in G6PDn individuals with CYP phenotypes and genotypes.

Characteristics	*CYP2C19* (N= 5)				*CYP2D6* (N= 5)				*CYP3A4* (N= 5)		
	**gNM**	**gIM**	**gRM**	**p value**	**gNM**	**gIM**	**gUM**	**p value**	***1/*1**	***1/*1B**	**p value**
Wild type, n/N (%)	4 (100.0)	-	1 (100.0)	-	2 (100.0)	3 (100.0)	-	-	2 (100.0)	3 (100.0)	-
No anemia (Hb), n/N (%)	1 (25.0)	-	-	>0.999	-	1 (33.3)	-	>0.999	1 (50.0)	-	>0.999
Mild anemia (Hb), n/N (%)	1 (25.0)	-	-		-	1 (33.3)	-		-	1 (33.3)	
Moderate Anemia (Hb), n/N (%)	1 (25.0)	-	1 (25.0)		1 (33.3)	1 (33.3)	-		1 (50.0)	1 (33.3)	
Severe anemia (Hb), n/N (%)	1 (25.0)	-	-		1 (33.3)	-	-		-	1 (33.3)	
TB (mg/dL), median (IQR)	2.3 (1.5-12.1)	-	0.9 (-)	0.1797	6.5 (0.9-12.1)	1.9 (1.5-2.3)	-	>0.999	1.2 (0.9-1.5)	7.2 (2.3-12.1)	0.1213
DB (mg/dL), median (IQR)	1.0 (0.5-7.0)	-	0.3 (-)	0.1797	3.6 (0.3-7.0)	0.7 (0.5-1.0)	-	>0.999	0.4 (0.3-0.5)	4.0 (1.0-7.0)	0.1213
IB (mg/dL), median (IQR)	1.3 (1.0-5.1)	-	0.6 (-)	0.1797	2.8 (0.6-5.1)	1.1 (1.0-1.3)	-	>0.999	0.8 (0.6-1.0)	3.2 (1.3-5.1)	0.1213
SGOT (U/L), median (IQR)	45.5 (32-94)	-	33 (-)	0.4795	82.5 (33-132)	35 (29-56)	-	0.5637	44.5 (33-56)	35 (29-132)	>0.999
SGPT (U/L), median (IQR)	33.5 (28-112.5)	-	36 (-)	>0.999	112 (36-188)	30 (26-37)	-	0.2482	33 (30-36)	37 (26-188)	0.5637
LDH (U/L), median (IQR)	601 (414-1,613)	-	-	-	1613 (-)	507.5 (414-601)	-	0.2207	-	601 (414-1,613)	-
Glucose (mg/dL), mean (SD)	147 (-)	-	-	-	-	147 (-)	-	-	-	147 (-)	-
Creatinine (mg/dL), median (IQR)	1.3 (0.45-2.3)	-	0.4 (-)	0.2765	0.4 (0.4-0.5)	2.2 (0.4-2.4)	-	0.3743	0.4 (-)	2.2 (0.5-2.4)	0.0756

Urea (mg/dL), median (IQR)	42.5 (31.5-59)	-	20 (-)	0.1573	28 (20-36)	49 (27-69)	-	0.2482	23.5 (20-27)	49 (36-69)	0.0833
Hemoglobinuria, n/N (%)	1 (100.0)	-	-	-	-	1 (100.0)	-	-	-	1 (100.0)	-
Proteinuria, n/N (%)	1 (100.0)	-	-	-	-	1 (100.0)	-	-	-	1 (100.0)	-
Bilirubinuria, n/N (%)	-	-	-	-	-	-	-	-	-	-	-

Abbreviations: Wild type= G6PDn, Hb= haemoglobin, TB= total bilirubin, DB= direct bilirubin, IB= indirect bilirubin, LDH= lactic dehydrogenase, gNM= normal metabolizer, gIM= intermediate metabolizer, gRM= rapid metabolizer, gUM= ultra-rapid metabolizer, SD= standard deviation, IQR= interquartile range. Symbol * refers to the star allele.
